# Defining the Minimal Clinically Important Difference (MCID) and Patient Acceptable Symptom State (PASS) at 2 years following open gluteus medius and/or minimus repair

**DOI:** 10.1093/jhps/hnad019

**Published:** 2023-07-26

**Authors:** Morgan W Rice, Robert B Browning, Thomas W Fenn, Mario Hevesi, Shane J Nho

**Affiliations:** Section of Young Adult Hip Surgery, Division of Sports Medicine, Department of Orthopedic Surgery, Rush Medical College of Rush University, Rush University Medical Center, 1611 W. Harrison St., Chicago, IL 60612, United States; Section of Young Adult Hip Surgery, Division of Sports Medicine, Department of Orthopedic Surgery, Rush Medical College of Rush University, Rush University Medical Center, 1611 W. Harrison St., Chicago, IL 60612, United States; Section of Young Adult Hip Surgery, Division of Sports Medicine, Department of Orthopedic Surgery, Rush Medical College of Rush University, Rush University Medical Center, 1611 W. Harrison St., Chicago, IL 60612, United States; Section of Young Adult Hip Surgery, Division of Sports Medicine, Department of Orthopedic Surgery, Rush Medical College of Rush University, Rush University Medical Center, 1611 W. Harrison St., Chicago, IL 60612, United States; Section of Young Adult Hip Surgery, Division of Sports Medicine, Department of Orthopedic Surgery, Rush Medical College of Rush University, Rush University Medical Center, 1611 W. Harrison St., Chicago, IL 60612, United States

## Abstract

To define Minimally Clinically Important Difference (MCID) and Patient Acceptable Symptomatic State (PASS) threshold scores after open gluteus medius and/or minimus repair. Primary open gluteus medius and/or minimus repair patients from November 2013 to March 2020 were identified. Patient reported outcomes (PROs) were assessed preoperatively, 1- and 2-year follow-up, including the Hip Outcome Score Activities of Daily Living (HOS-ADL), modified Harris Hip Score (mHHS), International Hip Outcome Tool-12 (iHOT-12) and Visual Analog Scale (VAS) Pain. Thresholds for achieving a MCID and PASS postoperatively were calculated using the distribution method and receiver operator curve analysis; 25 patients (24 females, 1 male, age: 69 ± 6.8 years, body mass index: 26.9 ± 5.0 kg/m^2^) were included in final analyses. MCID threshold scores for HOS-ADL, mHHS, iHOT-12 and VAS Pain were calculated as 11.1, 6.2, 15.3 and 14.0, respectively. PASS threshold scores for each of the PROs were as follows: HOS-ADL (71.9), mHHS (60.0), iHOT-12 (49.2) and VAS Pain (36.8). MCID thresholds for HOS-ADL, mHHS, iHOT-12 and VAS Pain were achieved by 58.3%, 83.3%, 66.7% and 57.1% of patients, respectively. PASS thresholds for HOS-ADL, mHHS, iHOT-12 and VAS Pain were achieved by 52.4%, 44.8%, 65% and 59.1% of patients, respectively. Open gluteus medius and/or minimus repair results in a high rate of achievement of clinically significant outcomes at a minimum of 2 years postoperatively. MCID threshold values for HOS-ADL, mHHS, iHOT-12 and VAS Pain were 11.1, 6.2, 15.3 and 14.0, respectively. PASS threshold values for HOS-ADL, mHHS, iHOT-12 and VAS Pain were 71.9, 60.0, 49.2 and 36.8, respectively. The majority of patients achieved clinically significant outcomes with 81.3% and 77.3% achieving MCID and PASS for at least one PRO, respectively.

## INTRODUCTION

The hip abductor muscle group consists of the gluteus medius, gluteus minimus and tensor fasciae latae located on the lateral hip and thigh, with increasing prevalence in an older female population [1, 2]. Both open and endoscopic repair techniques have proven viable treatment options for gluteus medius/minims (GM) tears, with more recent studies directly comparing the two techniques [3]. Following open GM repair, multiple studies have demonstrated improvements in patient reported outcomes (PROs) at short-term follow-up with similar improvement to that of endoscopic repair [3–8]. After open repair, significant improvements in modified Harris Hip Score (mHHS), from 67 points preoperatively to 85 points at 2 years, have been demonstrated, comparable to that of endoscopic repairs, with improvements from 50 points preoperatively to 84 points at 2 years [4, 6, 7]. A review by Chandrasekaran *et al.* of three open and four endoscopic repair outcome studies found both approaches resulted in significant improvements in multiple PROs [5]. The study was limited in that no direct comparisons were made between approaches due to the lack and heterogeneity of overlapping PROs between the open and endoscopic studies. It is also noteworthy that no comparisons of achievement of clinically significant outcomes (CSOs) including Minimally Clinically Important Difference (MCID) or Patient Acceptable Symptomatic State (PASS) were made between techniques, particularly given that for many PROs, MCID and PASS thresholds have yet to be established for open repair techniques.

With a growing emphasis on CSOs, it is important to define unique threshold scores specific to surgical approach in order to facilitate more accurate comparisons. To date, few studies have defined threshold scores to achieve MCID or PASS for open repairs. Meghpara *et al.* calculated MCID thresholds for the mHHS, Non-Arthritic Hip Score (NAHS) and Hip Outcome Score—Sports Subscale (HOS-SS) to be 7.5, 7.4 and 10.9, respectively, with 79.3%, 86.4% and 70.2% of patients achieving the MCID for each respective PRO [9]. The study included both endoscopic and open repairs with a large majority of patients having been treated with an endoscopic approach, limiting the generalizability to isolated open repairs [9]. Maldonado *et al.* defined MCID and PASS thresholds for the mHHS after open repair of full thickness GM tears [10]. However, the authors included cases in which additional intra-articular pathology was addressed with concomitant hip arthroscopy, potentially biasing results with interventions in anatomic locations other than the gluteal musculature [10].

Currently, no published studies have defined MCID or PASS thresholds for the Hip Outcome Score—Activities of Daily Living (HOS-ADL), International Hip Outcome Tool-12 (iHOT-12) and Visual Analog Scale (VAS) for Pain following open isolated gluteus medius and/or minimus repair. Therefore, the purpose of this study was to define 2-year postoperative MCID and PASS threshold scores after open GM repair.

## METHODS

### Patient selection

After obtaining institutional review board approval (Rush University Medical Center), a retrospective review of a prospectively maintained, single institutional database was conducted to identify patients who underwent primary open GM repair by the senior author from November of 2013 through March 2020 and with a minimum of 2-year follow-up. Patients were included if they had clinical and imaging-based diagnosis of GM tear, failure of nonoperative treatment (NSAIDs, physical therapy, or trochanteric injections), subsequent surgical repair and had completed at least one PRO measure at a minimum 2 years postoperatively. Exclusion criteria included previous ipsilateral GM repair, those undergoing concomitant hip procedures, history of ipsilateral hip disorder (eg, avascular necrosis, slipped capital femoral epiphysis, or Legg-Calvé-Perthes) or operative reports unavailable for review. A total of 40 eligible patients were identified and 25 met the inclusion and exclusion criteria.

### Data collection and statistical analysis

Patient demographics including age, gender and body mass index (BMI) were prospectively collected in a secure repository. Additional preoperative characteristics including smoking status, physical activity status, workers compensation status, history of low-back pain and psychiatric history were obtained. Preoperative magnetic resonance imaging (MRI) was reviewed and assessed for fatty infiltration and graded according to the Goutallier–Fuchs (G-F) classification system [11]. The size of partial thickness tears as a percentage of the total tendon footprint and degree of retraction in centimeters from the tendon footprint was also assessed using MRI. Intraoperative findings and procedures were recorded including whether tears were partial or full-thickness or involving the gluteus medius, gluteus minimus or both. Continuous variables were reported as averages with standard deviations. Categorical variables were reported as percentage of the total study population. Paired sample *t*-tests were utilized to compare PROs between preoperative, 1-year and 2-year time-points. An alpha level of 0.05 was used to determine statistical significance. All data analyses were completed using SPSS, version 28.0 (IBM, Armonk, NY, USA).

### Surgical technique

**Fig. 1.**
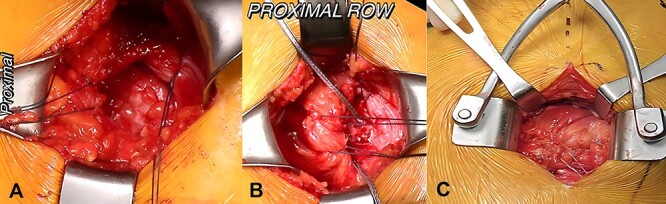
Open primary gluteus medius and minimus repair with (A) visualization and demonstration of tear mobility utilizing traction sutures, (B) placement of two proximal row anchors and traction sutures to mobilize tear, (C) completion of four anchor (2 proximal anchors, 2 distal anchors) repair.

All cases were performed under general endotracheal anesthesia with the patient in the lateral decubitus position and the operative leg prepped and draped in a standard surgical fashion. An incision was made centered over the greater trochanter. Deep dissection was conducted to the level of the IT band and the IT band was incised in line with fibers. The trochanteric bursa was visualized and debrided. GM pathology was then directly visualized and free tendinous ends were tagged (Fig. 1A). A burr was used to decorticate the greater trochanter in the location of the abductor footprint to a bleeding bed of bone. The torn GM tendon was mobilized to the greater trochanter to allow for a tension-free repair.

Once mobilized, the first 4.75 mm PEEK suture anchor (Corkscrew, Arthrex, Naples, FL, USA) anchor was placed at the superoposterior aspect of the tendon footprint, followed by a second anchor that was placed anterior to this one, creating a proximal row (Fig. 1B). Free suture ends from the proximal anchors were then passed through the torn gluteal tendons and tied in a horizontal mattress configuration. When the proximal row was secured, attention was turned toward the distal row. A second set of two knotless 4.75 mm suture anchors (SwiveLock, Arthrex, Naples, FL, USA), one anterior and one posterior, were placed and the sutures were passed through the tendon and secured, creating a double row suture configuration. The construct was then evaluated through a range of hip motions to ensure that the repair provided excellent biomechanical fixation of the torn tendons and the IT band was closed (Fig. 1C). Sterile dressings were applied. All patients followed a standard postoperative rehabilitation protocol [12].

### Clinically significant outcomes analysis

Patient reported outcomes were prospectively collected including HOS-ADL, mHHS, iHOT-12 and VAS Pain preoperatively, 1 year and 2 years postoperatively using secure electronic data collection platforms. Additionally, VAS for Satisfaction was collected at 1 and 2 years postoperatively. Clinically significant outcomes were defined by the achievement of or failure to achieve an MCID or PASS threshold score at 2 years postoperatively. MCID is defined as the minimal amount of improvement between preoperative and postoperative PRO scores correlating to a minimal amount of clinical improvement in function which patients’ perceive as beneficial in the absence of troublesome side effects and excessive cost [13, 14]. PASS is defined by achievement of a minimum threshold score above which a patient has achieved a satisfactory symptom state [15].

To determine unique MCID thresholds for each PRO, the distribution method was used by taking one half the standard deviation of the difference between preoperative and 2 years postoperative PRO scores [16]. Once a threshold was calculated, individual patient’s preoperative and 2-year postoperative difference was calculated and compared to the unique MCID threshold score to determine achievement.

Unique PASS thresholds for each PRO were calculated using a receiver operator curve (ROC) analysis with Youden’s index. At the 2-year postoperative timepoint, patients were asked the following yes or no question: ‘Taking into account all the activities you have during your daily life, your level of pain, and also your functional impairment, do you consider that your current state is satisfactory?’ The answer to this question was then coded as the binary outcome (0 = no, 1 = yes) for the ROC analysis. The AUC–ROC was analyzed to determine the overall performance of the binary classifier, with a minimum AUC of 0.7 to be considered acceptable discriminative ability [17–19]. Once calculated, Youden’s index (calculated as sensitivity + sensitivity) was utilized to maximize the sensitivity and specificity of the threshold score along the point coordinates of the ROC curve. The point with the greatest Youden’s index was determined as the threshold score. A patient’s individual 2-year PRO score was compared against the unique PASS threshold to determine rate of achievement. Incomplete or missing PRO surveys were excluded from analysis.

## RESULTS

### Study population

Twenty-five patients (24 females, 1 male) who underwent primary open gluteus medius and/or minimus repair were included at final analysis at a mean follow-up of 3.1 years (range 2.5–5.4 years) (Table I). Average preoperative pain duration was 19.6 ± 24.7 months (range: 1–102 months). Thirteen patients (52.0%) reported they were physically active. One patient (4.0%) had a history of anxiety or depression. Two patients (8.0%) were current or former smokers. Fourteen patients (56.0%) had a history of low-back pain. No workers compensation patients were included.

**Table I.** Patient demographics and preoperative characteristics

	Open gluteus medius and/or minimus repair
*N*	25
Age, years	69.0 ± 6.8
Sex, % female	96.0%
Body mass index, kg/m^2^	26.9 ± 5.0
Smoking, current or former	8.0%
Physically active	52.0%
Prior ipsilateral THA	4.0%
Workers compensation	0.0%
Low-back pain	56.0%
Psychiatric history	4.0%
Preoperative pain duration (mos.)	19.6 ± 24.7

Abbreviations: THA, total hip arthroplasty; mos, months.

### MR imaging, intraoperative findings and procedures

Preoperative MRIs were available and reviewed for 92.0% of patients (*n* = 23). For those patients without preoperative MRIs available for review (*n* = 1 in setting of prior THA, *n* = 1 with hip pinning in the setting of prior femoral neck fracture), intraoperative assessment was used to identify the extent of tearing and tendon involvement. A majority of patients 68.0% (*n* = 17) demonstrated full thickness tearing. Both the gluteus medius and minimus was involved in a majority of cases 80.0% (*n* = 20), with 5 and 0 of the patients having isolated gluteus medius or minimum tears, respectively. Assessment of fatty infiltration according to the G-F classification system demonstrated four patients (17.4%) with Grade 1 fatty infiltration, seven patients (30.4%) with Grade 2, seven patients (30.4%) with Grade 3 and five patients (21.7%) with Grade 4 (Table II).

**Table II.** Preoperative MRI findings

	Open hip abductor repair
Tendons involved, *n* (%)	
Gluteus medius	5 (20.0%)
Gluteus minimus	0 (0.0%)
Both	20 (80.0%)
Goutallier–Fuchs, *n* (%)	
Grade 1	4 (19.1%)
Grade 2	7 (23.8%)
Grade 3	7 (33.3%)
Grade 4	5 (23.8%)
Full thickness, *n* (%)	17 (68.0%)
Retraction, mm	21.4
Partial thickness, *n* (%)	8 (32.0%)
Tear size, %	50.7%
Retraction, mm	7.5

Abbreviations: mm, millimeter; *n*, number.

### Postoperative outcomes analysis

Three patients underwent revision gluteus medius/minimus surgery at an average of 5.4 months (range: 2.1–7.4 months) and were excluded from subsequent PRO analysis. Overall, 2-year revision-free survivorship was 88%. Two patients underwent revision open GM repair with superior gluteal reconstruction (SGR) with an acellular dermal allograft at 2.1 and 7.4 months due to traumatic re-tearing of both the gluteus medius and minimus. The third patient underwent revision open GM repair with SGR with an acellular dermal allograft and concurrent total hip arthroplasty revision at 6.8 months due to retearing after recurrent hip dislocations. Re-tearing was confirmed on repeat MRI prior to revision in all cases.

Primary open GM repair resulted in significant improvement in all PRO measures at 1 and 2 years postoperatively. No significant changes were seen in any PRO from 1 to 2 years postoperatively (Table III).

**Table III.** Patient reported outcome scores at baseline preoperatively, 1 year and 2 years postoperatively

				*P*-value		
	Preoperative	1 year postoperative	2 year postoperative	Preop vs 1 year	Preop vs 2 year	1 year vs 2 year
HOS-ADL	52.0 ± 16.9	66.9 ± 21.4	67.3 ± 26.1	<0.001*	0.006*	0.146
mHHS	49.5 ± 11.9	60.2 ± 13.0	63.3 ± 23.4	0.003*	<0.001*	0.882
iHOT-12	22.1 ± 15.1	50.1 ± 21.7	56.2 ± 31.0	0.002*	<0.001*	0.805
VAS Pain	63.3 ± 24.5	34.9 ± 12.7	36.4 ± 29.4	<0.001*	<0.001*	0.477
VAS Satisfaction		71.4 ± 18.6	77.5 ± 24.6			0.400

Abbreviations: HOS-ADL, Hip Outcome Score Activities of Daily Living subscale; mHHS, modified Harris Hip score; iHOT-12, International Hip Outcomes Tool-12; VAS Pain/Satisfaction, Visual Analog Scale for Pain and Satisfaction. *Indicates statistically significant result at an alpha level of <0.05.

### MCID and PASS thresholds

Unique MCID thresholds for HOS-ADL, mHHS, iHOT-12 and VAS Pain were calculated, with a majority of patients achieving the MCID threshold for each PRO (Table IV). Unique PASS thresholds for HOS-ADL, mHHS, iHOT-12 and VAS Pain were calculated, all of which demonstrated acceptable discriminative ability, as well as high rates of achievement (Table V).

**Table IV.** Unique MCID threshold scores and achievement for 2 year PROs

	MCID threshold	Achievement
HOS-ADL	11.1	58.3%
mHHS	6.2	83.3%
iHOT-12	15.3	66.7%
VAS Pain	14.0	57.1%

Abbreviations: HOS-ADL, Hip Outcome Score Activities of Daily Living subscale; mHHS, modified Harris Hip score; iHOT-12, International Hip Outcomes Tool-12; VAS Pain/Satisfaction, Visual Analog Scale for Pain.

**Table V.** Unique PASS threshold scores, achievement, sensitivity, specificity and AUC for 2 year PROs

	PASS threshold	AUC	Sensitivity	Specificity	Achievement
HOS-ADL	71.9	0.74	75.0%	75.0%	52.4%
mHHS	60	0.76	76.9%	62.5%	44.8%
iHOT-12	49.2	0.97	92.3%	85.7%	65.0%
VAS Pain	36.8	0.74	75.0%	71.4%	59.1%

Abbreviations: HOS-ADL, Hip Outcome Score Activities of Daily Living subscale; mHHS, modified Harris Hip score; iHOT-12, International Hip Outcomes Tool-12; VAS Pain/Satisfaction, Visual Analog Scale for Pain; PASS, Patient Acceptable Symptom State; AUC, area under the curve.

A majority of patients achieved positive clinical outcomes with 81.3% of patients achieving MCID for at least at least one PRO, 77.3% of patients achieving PASS for at least one PRO and 73.3% achieving both MCID and PASS for at least one PRO. When assessing each PRO, high rates of achievement were seen for both MCID and PASS for each PRO assessed (Table VI). For patients with MCID and PASS scores for all four PRO outcomes, 50% achieved MCID for all outcomes, 27.8% achieved PASS for all outcomes and 33.3% achieved both MCID and PASS for all outcomes.

**Table VI.** Achievement of 2 year clinically significant outcomes for each PRO score

	Achieved both MCID/PASS	Achieved MCID/not PASS	Achieved PASS/not MCID	Achieved neither PASS/MCID
HOS-ADL	41.7%	16.7%	8.3%	33.3%
mHHS	58.3%	25.0%	0.0%	16.7%
iHOT-12	50.0%	8.3%	8.3%	33.3%
VAS Pain	50.0%	7.1%	14.3%	28.6%

Abbreviations: MCID, Minimally Clinically Important Difference; PASS, Patient Acceptable Symptomatic State; HOS-ADL, Hip Outcome Score Activities of Daily Living subscale; mHHS, modified Harris Hip score; iHOT-12, International Hip Outcomes Tool-12; VAS Pain, Visual Analog Scale for Pain.

## DISCUSSION

The purpose of the present study was to define MCID and PASS thresholds at a minimum of 2 years postoperatively for patients undergoing isolated open GM repair. Open repair resulted in statistically significant improvement at both 1 and 2 years postoperatively in all PROs. Additionally, we defined unique 2-year MCID and PASS thresholds for HOS-ADL, mHHS, iHOT-12 and VAS Pain, with high rates of achievement for both. The majority of patients achieved CSOs with 81.3% of patients achieving MCID and 77.3% achieving PASS for at least PRO. Additionally, 73.3% of patients achieved both MCID and PASS for at least one PRO.

There has been a shift in orthopedic literature toward defining CSOs to better contextualize statistically significant outcomes following surgical intervention. CSOs following endoscopic GM repair have been defined at both short- and mid-term follow-up with improvement following repair well documented within the literature [5, 19–23]. Despite this, no studies to date have defined MCID and PASS at a minimum of 2 years postoperatively for isolated open GM repair for HOS-ADL, mHHS, iHOT-12 or VAS Pain.

Meghpara *et al*. [9] and Maldonado *et al.* [10] defined MCID and PASS threshold scores for a variety of different PROs at 2 years postoperatively, with excellent achievement rates of these clinically significant outcomes. Both of these studies are limited to their generalizability, as heterogeneous patient populations were included. Meghpara *et al.* [9] cohort included >90% of patients undergoing endoscopic repair, limiting the use in defining CSOs following open repair. Maldonado *et al.* [10] included patients undergoing open repair, however, nearly one third of the patients underwent an arthroscopic procedure for concomitant intra-articular pathology, thus, limiting the generalizability to patients who undergo isolated open repair. Uppstrom *et al*. [8] defined MCID for mHHS (9.9) and iHOT-33 (14.3) for patients undergoing open repair at a minimum of 10 months postoperatively and demonstrated a high rate of achievement in both PROs. Despite this, many of the PROs in Uppstrom *et al.* [8] were obtained prior to 2 years with a minimum 10-month follow-up.

Okoroha *et al.* [19] defined MCID and PASS thresholds for patients 2 years after isolated endoscopic GM repair. They defined MCID for HOS-ADL, HOS-SS, mHHS to be 15.02, 14.53 and 14.13, respectively, and defined PASS for the same PROs as 77.9, 56.9 and 69.3, respectively. The present study defined MCID threshold values for HOS-ADL, mHHS, iHOT-12 and VAS Pain as 11.1, 6.2, 15.3 and 14.0, respectively. Additionally, the PASS threshold values for HOS-ADL, mHHS, iHOT-12 and VAS Pain were found to be 71.9, 60.0, 49.2 and 36.8, respectively. In comparison to the thresholds defined for endoscopic repair, the values for the achievement of MCID following open repair in this study were less for the mHHS and HOS-ADL scores [19]. The threshold values for achievement of PASS following open repair was also lower for the mHHS and HOS-ADL scores. Although the study by Okohora *et al*. did not describe preoperative tear characteristics, the differences in threshold values can likely be attributed to more severe tear characteristics seen in patients who undergo open repair versus those who undergo endoscopic repair.

Nearly, 70% of tears in the present study were full thickness tears with an average tear retraction of >2 cm. The influence of preoperative tear characteristics on clinical decision making and outcomes following repair is well documented throughout the literature. A systematic review by Looney *et al*. assessed the role of fatty infiltration on outcomes following gluteus medius/minimus repair in four studies and concluded that patients with high grade fatty infiltration (Grade 3 or 4 G-F) may benefit from open repair [24]. Additionally, size of preoperative gluteus medius/minimus thickness tears (partial versus full) has demonstrated successful postoperative outcomes after repair, however, with lower functional improvement for full-thickness tears [11, 23]. Given the high prevalence of full thickness tears in this cohort, this may be similarly impacting outcomes compared to the endoscopic only studies. This notion provides an exciting opportunity for future research to further elucidate the impact on how tear characteristics impact postoperative outcomes and to further correlate these to preoperative MRI findings. As demonstrated by the significant 2-year PRO improvement in this investigation and high rate of MCID and PASS achievement, open repair remains an important treatment modality for patients with intractable lateral hip pain and tears with significant retraction, fatty infiltration or full thickness. The present study found that 81.3% and 77.3% achieved at least one MCID and one PASS threshold score, respectively, which is similar to that seen following endoscopic repair (78% and 69%, respectively), which furthers the notion that open repair is a viable treatment option [19].

## LIMITATIONS

The present study is not without important limitations. First, although this study is one of the largest series of open GM repairs with a minimum of 2-year follow-up, our cohort is still limited to a small sample size of 25 patients. A larger sample size would allow for more advanced statistical methodology to determine predictors of MCID and PASS. Second, its retrospective design is inherently vulnerable to biases. An attempt was made to mitigate selection bias by including all consecutive cases during the study period. Third, the lack of endoscopic control group or randomization limits its ability to make direct comparisons to endoscopic repairs. Further investigation through a prospectively enrolling study would be needed to definitively compare the differences in tear characteristics and outcomes of open and endoscopic approach techniques. Fourth, patients did not undergo postoperative MRI imaging unless indicated by possible failure or retear. MRI completion postoperative would strengthen the study to evaluate for integrity of repair and correlation with clinical outcomes. Lastly, our study population consisted of consecutive GM tears repaired by a single, high-volume, hip arthroscopist at a major academic medical center, which may limit our ability to provide generalizable conclusions.

## CONCLUSION

In conclusion, open gluteus medius and/or minimus repair results in a high rate of achievement of clinically significant outcomes at a minimum of 2 years post-operatively. MCID threshold values for HOS-ADL, mHHS, iHOT-12 and VAS Pain were 11.1, 6.2, 15.3 and 14.0, respectively. PASS threshold values for HOS-ADL, mHHS, iHOT-12 and VAS Pain were 71.9, 60.0, 49.2 and 36.8, respectively. The majority of patients achieved clinically significant outcomes with 81.3% and 77.3% achieving MCID and PASS for at least PRO, respectively.

## DATA AVAIILABILITY

The data underlying this article will be made upon request to the corresponding author.
